# Associations between dietary magnesium intake and handgrip strength were modified by serum vitamin D level among the US elderly

**DOI:** 10.3389/fnut.2022.1002634

**Published:** 2022-10-12

**Authors:** Shuang Han, Yue Gao, Da Gan

**Affiliations:** ^1^Department of Geriatrics, Affiliated Hangzhou First People's Hospital, Zhejiang University School of Medicine, Hangzhou, China; ^2^Chronic Disease Research Institute, The Children's Hospital, and National Clinical Research Center for Child Health, School of Public Health, School of Medicine, Zhejiang University, Hangzhou, China; ^3^Department of Nutrition and Food Hygiene, School of Public Health, School of Medicine, Zhejiang University, Hangzhou, China

**Keywords:** magnesium intake, handgrip strength, sarcopenia, serum vitamin D, older people

## Abstract

**Objective:**

The present study aimed to evaluate the associations between dietary magnesium intake and handgrip strength, and whether these associations were affected by serum vitamin D status.

**Methods:**

A total of 2,127 participants aged 60 and above from the National Health and Nutrition Examination Survey (NHANES) of the 2011–2014 cycles were included in the analyses. Magnesium intake was obtained by 24-h dietary recalls and 30-day dietary supplement. Participants in the lowest sex-specific tertile of magnesium intake were defined as having low magnesium intake. Serum 25-hydroxyvitamin D [25(OH)D)] concentrations were examined by using ultra-high performance liquid chromatography tandem mass spectrometry and categorized into three levels: deficient, suboptimal, and sufficient. Handgrip strength was determined by using a dynamometer. Multivariable linear regression models were used to investigate the associations between dietary magnesium intake and handgrip strength.

**Results:**

Low magnesium intake was not associated with handgrip strength, but interactions between low magnesium intake and serum 25(OH)D level existed on handgrip strength. The stratified analyses found that only in participants with deficient serum 25(OH)D, low magnesium intake was associated with reduced handgrip strength. The combined analyses shown that participants with both low magnesium intake and deficient serum 25(OH)D had highest decrease of handgrip strength.

**Conclusion:**

Findings suggested that low magnesium intake was associated with reduced handgrip strength only in participants with deficient serum 25(OH)D. Increased magnesium intake was recommended for participants with deficient serum 25(OH)D in maintaining muscle strength.

## Introduction

Sarcopenia is a geriatric syndrome characterized by progressive loss of muscle mass, muscle strength, and physical performance with aging (1). As a key component of sarcopenia, weak muscle strength was associated with increased risk of fracture, mobility disorders, and mortality (2–4). Findings reported that more than half of the U.S. elderly suffered from muscle weakness (5), which highlight the importance of adopting effective interventions in preventing muscle weakness.

It is known that diet intervention, especially increased intake of dietary protein, is one of the effective strategies in improving muscle strength (6). However, the role of dietary magnesium intake in muscle strength is not well understood (7–12). Magnesium is an essential element in human, and participates in various physiological process including energy metabolism, electrolyte balance, and muscle contraction and relaxation (13), all of which were closely related to muscle strength. To date, only a few studies explored the associations between dietary magnesium intake and muscle strength, and the results were inconsistent (7–12).

In addition, studies found that magnesium could affect the synthesis and metabolism of vitamin D (14), and meanwhile vitamin D could influence the absorption of magnesium (15, 16). Interactive effect between magnesium intake and serum vitamin D status was observed on hypertension, diabetes and cardiovascular mortality (17–19). Considering the effect of vitamin D on muscle strength (20), whether interaction between magnesium intake and vitamin D also existed on muscle strength is unclear.

Hence, the present study aimed to investigate the associations between dietary magnesium intake and muscle strength, and whether these associations were influenced by serum vitamin D levels.

## Methods

### Study participants

The National Health and Nutrition Examination Survey (NHANES) used a stratified, multistage, probability cluster sampling design to assess the nationally representative health and nutritional status of the non-institutionalized US civilian. Because sarcopenia mostly happened in the elderly, our study included data from 3,632 adults aged 60 and above in the NHANES (2011–2012 and 2013–2014). We further excluded participants with missing data on magnesium intake (*N* = 825), serum 25-hydroxyvitamin D [25(OH)D] (*N* = 131), handgrip strength (*N* = 249), and potential cofounders (*N* = 300), resulting in a final analytic sample of 2,127 participants.

The NHANES procedures and protocols were approved by the National Center for Health Statistics Research Ethics Review Board. The written informed consent was obtained from each adult participant (21).

### Assessments of magnesium intake

Data regarding dietary intake were collected *via* two 24-h recalls. The first dietary recall was conducted in the mobile examination centers (MEC), and the second dietary recall was completed by trained interviewers *via* telephone 3–10 days after the MEC interview (22). The consumption of all foods and beverages during the past 24 h were recalled by participants and were recorded by interviewers using the United States Department of Agriculture (USDA) automated multiple-pass method (23). Then, these data were used to determine dietary energy intake and nutrients intake (including protein, magnesium, calcium and so on) by using the USDA Food and Nutrient Database for Dietary Studies (FNDDS) (24). Data on the use of dietary supplements and non-prescription antacid were collected by using 30-day dietary supplement questionnaire (25).

Dietary magnesium intake was calculated as the average magnesium intake from the two 24-h recalls. The average amount of dietary magnesium supplement was calculated by summing all supplemental magnesium and dividing it by 30. Total magnesium intake was calculated as the sum of daily magnesium intake from diet and supplements. Due to differences in sex-specific magnesium intake recommendations (26), participants in the lowest sex-specific tertile of total magnesium intake were defined as having low magnesium intake (total magnesium intake <247.0 mg/day for men or <204.6 mg/day for women).

### Measurement and classification of serum 25(OH)D

Blood sample was drawn from participants and processed into vials in the MEC. These vials were stored under −30°C conditions until they were shipped to the National Center for Environmental Health for testing. The ultra-high-performance liquid chromatography-tandem mass spectrometry method (UHPLC-MS/MS) was used to examine the concentrations of serum 25-hydroxyvitamin D2 and D3 [25(OH)D2 and 25(OH)D3]. Total serum 25(OH)D was calculated as the sum of 25(OH)D2 and 25(OH)D3. According to the suggestions of US Endocrine Society, total serum 25(OH)D was categorized into deficient group (<50 nmol/L), suboptimal group (50–75 nmol/L), and sufficient group (>75 nmol/L) (27).

### Measurement of handgrip strength

Handgrip strength was evaluated using a Takei digital grip strength dynamometer (Model T.K.K.5401). Participants were asked to squeeze the dynamometer as hard as they could by using one hand in a standing position. And then the test was repeated for the other hand. Each hand was tested three times, alternating hands with a 60-s rest between measurements on the same hand. The combined grip strength, which was calculated as the sum of the largest reading from each hand, was used in our analyses (28).

### Covariates

Questionnaire survey were conducted to collect information on age, gender (male and female), race (Mexican American, other Hispanic, non-Hispanic White, non-Hispanic Black, and other race), marital status (never married, married or living with partner, and widowed or separated or divorced), education (less than high school, high school, and college education or above), family monthly poverty level index (PIR), smoke, alcohol drinking, and physical activity.

PIR is an index for the ratio of family monthly income to poverty, which was calculated as a ratio of monthly family income to the Department of Health and Human Services' poverty guidelines specific to family size. PIR was classified as three categories (PIR ≤ 1.3; 1.3 < PIR ≤ 1.85; PIR > 1.85) (29). Smoke was categorized into current (participants who smoked at least 100 cigarettes in life and now smoke), former (participants who smoked at least 100 cigarettes in life and quit smoke now), and never. Participants who had at least 12 alcohol drinks in any 1 year was classified as alcohol drinker. A modified Global Physical Activity Questionnaire was used to measure physical activity (30). The minutes spent per week engaging in different types of physical activity (including vigorous work-related activity, moderate work-related activity, walking or bicycling for transportation, vigorous leisure-time physical activity, moderate leisure-time physical activity) were multiplied by suggested metabolic equivalent (MET) scores of each type of physical activity, and then summed to calculate the total MET consumed per week (31). According to the median of total MET consumed by physical activity, physical activity was categorized into inactivate and activate. Body mass index (BMI) was classified as normal and underweight (<25 kg/m^2^), overweight (25.0–29.9 kg/m^2^), and obese (>30 kg/m^2^).

Similar to the calculation of total magnesium intake, total energy intake and total protein intake were calculated as the sum of energy intake and protein intake from diet and dietary supplement, respectively. Percentage of total energy from protein was calculated as total protein intake (gram) multiplied by 4 kcal/g and 100%, and then divided by total energy intake (kcal). Total calcium intake was the sum of calcium intake from diet and dietary supplement. Season of examination was categorized into November–April and May–October.

According to the AHA 2017 guidelines, participants were classified as having hypertension if participants taken any antihypertensive drugs, or had systolic blood pressure ≥130 mm Hg or diastolic blood pressure ≥80 mm Hg (32). Diabetes was defined if participants had self-reported diagnosis of diabetes, taken medicine for diabetes, or glycosylated hemoglobin ≥6.5%. Coronary heart disease (CHD) was ascertained if participants had history of CHD, angina or myocardial infarction. Stroke and cancer were determined if participants reported history of stroke or cancer (malignancy), respectively. The details of methodology and data collection are described elsewhere (33).

### Statistical analyses

Comparisons of baseline characteristics between participants with and without low magnesium intake were performed using weighted *t*-test and weighted chi-square test for continuous variables and categorical variables, respectively.

Weighted multivariable linear regression models were performed to investigate the associations between low magnesium intake and handgrip strength. Based on review of the previous studies and our clinical experience, age, gender, BMI group, race, marital status, education, PIR category, smoke, alcohol drinking, physical activity, energy intake, percentage of total energy from protein, calcium intake, hypertension, diabetes, CHD, stroke, cancer, and season of examination were adjusted in the regression models (28, 34, 35). Interactions between low magnesium intake and age, gender, BMI group, and serum 25(OH)D level were also examined. We also conducted stratified analyses to explore whether the associations between low magnesium intake and combined handgrip strength differed in different subgroups (age group, gender, BMI group, and serum 25(OH)D level). Finally, we investigated the combined associations of low magnesium intake and serum 25(OH)D level with handgrip strength.

All statistical analyses were performed using STATA 15.0. The *P*-value < 0.05 (two-sided) was considered statistically significant.

## Results

### Baseline characteristics of participants

Table 1 exhibited the baseline characteristics of participants with and without low magnesium intake. Compared to participants with normal magnesium intake, participants with low magnesium intake were more likely to be non-Hispanics Black, widowed or separated or divorced, and former smoker, and had higher BMI and higher prevalence of diabetes. In addition, participants with low magnesium intake had lower levels of education, PIR, physical activity, handgrip strength, energy intake, and calcium intake than without, and less likely to be alcohol drinker.

**Table 1:** Baseline characteristic of participants with and without low magnesium intake.

	**Normal magnesium intake (*n* = 1,417)**	**Low magnesium intake (*n* = 710)**	***P***
Age	69.0 (0.30)	69.5 (0.50)	0.369
Age categories			0.853
60–69	767 (57.3%)	358 (55.3%)	
70–79	424 (28.8%)	230 (29.5%)	
80+	226 (14.0%)	122 (15.2%)	
Gender (male)	693 (48.2%)	348 (43.0%)	0.152
Race			<0.001
Mexican American	123 (3.2%)	44 (3.5%)	
Other Hispanic	127 (3.6%)	66 (4.1%)	
Non-Hispanics white	782 (82.0%)	323 (74.3%)	
Non-Hispanics black	264 (6.2%)	226 (13.2%)	
Other race	121 (5.0%)	51 (5.0%)	
Marital status			0.008
Never married	76 (3.7%)	44 (3.8%)	
Married, living with partner	864 (69.0%)	381 (59.4%)	
Widowed, separated, or divorced	477 (27.3%)	285 (36.8%)	
Education			<0.001
Less than high school	271 (12.6%)	237 (24.0%)	
High school	323 (20.7%)	184 (26.4%)	
College education or above	823 (66.7%)	289 (49.7%)	
Family monthly poverty level index category			<0.001
≤ 1.3	354 (15.9%)	259 (26.6%)	
1.3–1.85	192 (11.4%)	114 (12.2%)	
>1.85	871 (72.7%)	337 (61.2%)	
Smoke			0.009
Never	704 (51.5%)	335 (49.2%)	
Former	139 (7.8%)	121 (13.8%)	
Current	574 (40.7%)	254 (37.0%)	
Alcohol drinking (yes)	1,022 (76.9%)	452 (64.3%)	<0.001
Physical activity			0.015
Inactive	678 (43.9%)	398 (54.5%)	
Active	739 (56.1%)	312 (45.5%)	
Body mass index (kg/m^2^)			<0.001
<25	386 (28.7%)	162 (21.2%)	
25.0–29.9	505 (37.4%)	244 (32.4%)	
≥30	526 (33.8%)	304 (46.4%)	
Handgrip strength (kg)	63.4 (0.95)	58.9 (1.27)	0.005
Energy intake (kcal)	2120.3 (22.9)	1365.8 (33.6)	<0.001
Percentage of total energy from protein (%)	16.1 (0.2)	16.4 (0.2)	0.257
Calcium intake (mg)	1039.0 (17.6)	603.1 (20.3)	<0.001
Serum 25(OH)D level (nmol/L)			0.064
Sufficient (>75)	760 (60.1%)	304 (50.3%)	
Suboptimal (50–75)	448 (29.0%)	222 (32.9%)	
Deficient (<50)	209 (10.9%)	184 (16.7%)	
Season of examination			0.902
November–April	629 (40.6%)	323 (41.3%)	
May–October	788 (59.4%)	387 (58.7%)	
Hypertension (yes)	1107 (76.2%)	581 (79.1%)	0.445
Diabetes (yes)	374 (20.6%)	260 (28.2%)	0.016
Coronary heart disease (yes)	209 (13.9%)	132 (18.4%)	0.101
Stroke (yes)	86 (5.6%)	70 (7.5%)	0.166
Cancer (yes)	299 (25.8%)	132 (22.1%)	0.244

Weighted means (standard errors) and counts (weighted percentages) are presented for continuous variables and categorical variables, respectively.

25(OH)D, 25-hydroxyvitamin D.

### Associations between low magnesium intake and handgrip strength

Table 2 shows the associations between low magnesium intake and handgrip strength. Low magnesium intake was not associated with handgrip strength (β, −0.20; 95% CI, −2.37–1.98). Significant interaction existed between low magnesium intake and serum 25(OH)D level (*P* = 0.044) on handgrip strength, while no such interactions between low magnesium intake and age, gender, and BMI group were observed.

**Table 2:** Associations between low magnesium intake and handgrip strength.

	**β (95% CI)**	***P***	***P* for interaction**
**Dietary magnesium intake**			
Normal magnesium intake	Referent		
Low magnesium intake	−0.20 (−2.37, 1.98)	0.851	
Age	−0.84 (−0.95,−0.72)	<0.001	0.693
**Gender**			
Male	Referent		
Female	−27.39 (−29.18, −25.60)	<0.001	0.067
**BMI group**			
Normal and underweight	Referent		
Overweight	3.20 (1.29, 5.11)	0.002	0.508
Obese	5.12 (3.34, 6.91)	<0.001	0.327
**Serum 25(OH)D level**			
Sufficient	Referent		
Suboptimal	−0.80 (−2.45, 0.85)	0.330	0.646
Deficient	−2.74 (−4.50, −0.98)	0.003	0.044

Adjusted for race, marital status, education, family monthly poverty level index category, smoke, alcohol drinking, physical activity, energy intake, percentage of total energy from protein, calcium intake, hypertension, diabetes, coronary heart disease, stroke, cancer, and season of examination.

β, beta coefficient; CI, confidence interval; 25(OH)D, 25-hydroxyvitamin D.

### Stratified associations between low magnesium intake and handgrip strength

**Figure 1:**
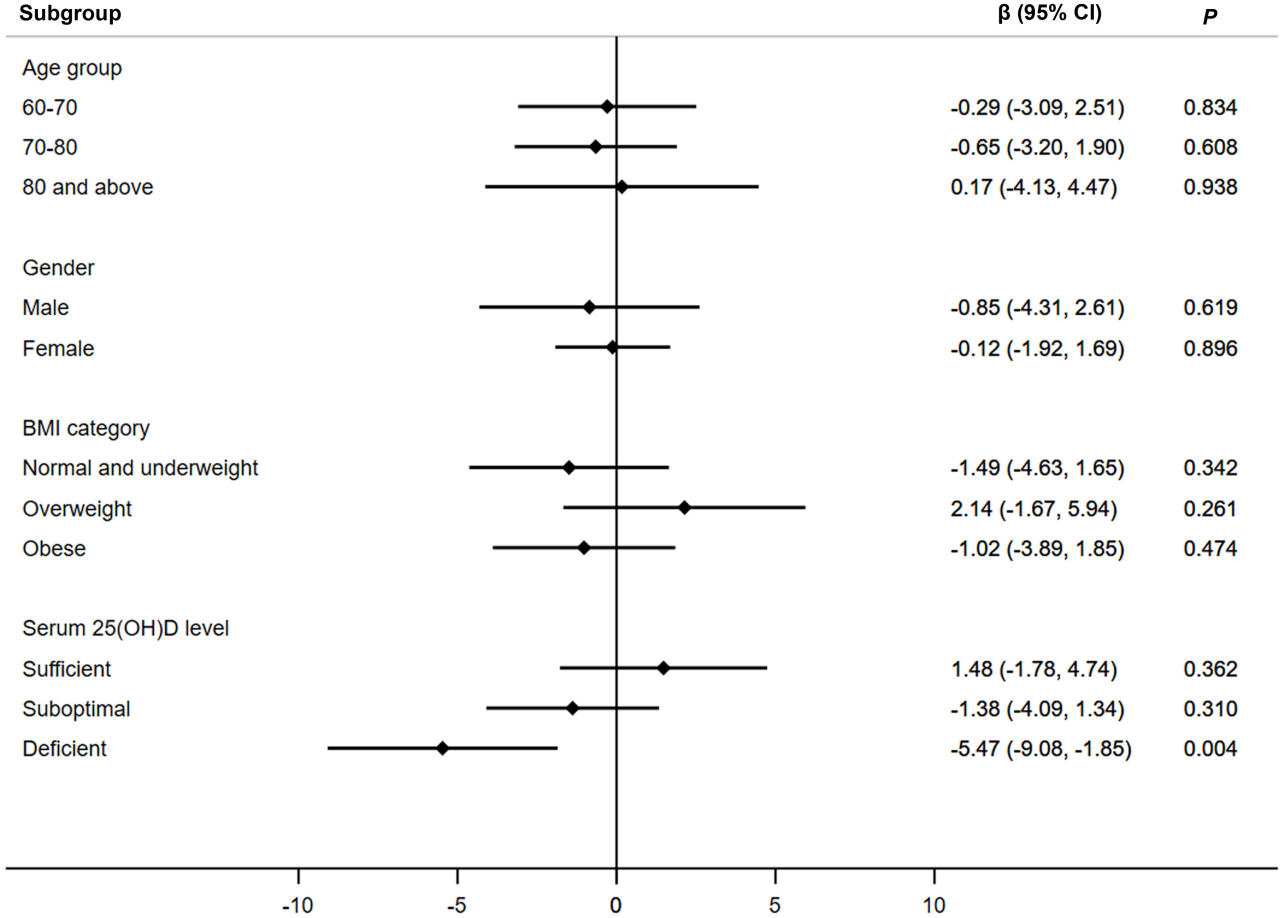
Associations between low magnesium intake and handgrip strength in different subgroups. Adjusted for age, gender, BMI group, serum 25(OH)D level, race, marital status, education, family monthly poverty level index category, smoke, alcohol drinking, physical activity, energy intake, percentage of total energy from protein, calcium intake, hypertension, diabetes, coronary heart disease, stroke, cancer, and season of examination. Age was not adjusted when participants were stratified by age groups. β, beta coefficient; CI, confidence interval; 25(OH)D, 25-hydroxyvitamin D.

Figure 1 presents the associations between low magnesium intake and handgrip strength in different subgroups. Only in participants with deficient serum 25(OH)D level, low magnesium intake was associated with reduced handgrip strength (β, −5.47; 95% CI, −9.08–−1.85). No significant associations were observed in other subgroups (participants in different age groups, gender, and BMI groups).

### Combined associations of magnesium intake and serum 25(OH)D with handgrip strength

Table 3 shows the combined associations of low magnesium intake and serum 25(OH)D level with handgrip strength. Compared with participants with normal magnesium intake and sufficient serum 25(OH)D, the handgrip strength decreased the most in participants with low magnesium intake and deficient serum 25(OH)D (β, −4.74; 95% CI, −7.34–−2.15).

**Table 3:** Combined associations of low magnesium intake and serum 25(OH)D level with handgrip strength.

	**Normal magnesium intake**		**Low magnesium intake**	
	**β (95% CI)**	***P***	**β (95% CI)**	***P***
Sufficient serum 25(OH)D	Referent		0.64 (−2.35, 3.64)	0.665
Suboptimal serum 25(OH)D	−0.60 (−2.61, 1.41)	0.548	−0.81 (−3.21, 1.59)	0.498
Deficient serum 25(OH)D	−1.37 (−3.83, 1.09)	0.264	−4.74 (−7.34, −2.15)	0.001

Adjusted for age, gender, BMI group, race, marital status, education, family monthly poverty level index category, smoke, alcohol drinking, physical activity, energy intake, percentage of total energy from protein, calcium intake, hypertension, diabetes, coronary heart disease, stroke, cancer, and season of examination.

β, beta coefficient; CI, confidence interval; 25(OH)D, 25-hydroxyvitamin D.

## Discussion

To the best of our knowledge, our study was the first to found that interactions existed between low magnesium intake and serum 25(OH)D level on handgrip strength, and low magnesium intake was associated with reduced handgrip strength only in participants with deficient serum 25(OH)D. In addition, the coexistence of both low magnesium intake and deficient serum 25(OH)D was associated with highest decrease of handgrip strength.

Low magnesium intake and serum vitamin D deficiency are two common phenomenons in the elderly. Approximately 70% of French adult and 68% of the US adults had magnesium intake less than the recommended daily allowance (36, 37), and the consumption of magnesium was further lower among the elderly. The vitamin D deficiency was reported to occur in more than a quarter of the US elderly (38). Therefore, clarifying the adverse effect of low magnesium intake, vitamin D deficiency and its combination on muscle strength is very meaningful. This might arouse people's attentions on magnesium intake and vitamin D deficiency, and help the elderly adopt effective interventions in preventing muscle weakness.

The associations between low magnesium intake and muscle strength were inconsistent (7–12, 39). Some previous studies found that high magnesium intake was associated with greater grip strength and magnesium supplement could enhance quadriceps strength (7–9), while other studies did not observe these phenomenons (10–12). These inconsistencies might be explained by the difference of participants' baseline characteristics, the inconsistent length of intervention, the different sample size of each study, and the different covariates in the regression models. Our study supported the view that low magnesium intake was not associated with handgrip strength in the whole elderly population. In addition, because muscle strength varied a lot by age group, gender and BMI groups, we performed stratified analyses and found that associations between low magnesium intake and handgrip strength remained insignificant in different age groups, gender, and BMI groups. Future studies with large sample size are needed to further elucidate the associations between magnesium intake and muscle strength.

Our studies observed the significant interaction between low magnesium intake and serum 25(OH)D level, and low magnesium intake was associated with reduced handgrip strength only in participants with deficient serum 25(OH)D. The reason for these findings might be that magnesium plays important roles in the metabolism of vitamin D (14). As a biologically inactive form in the circulation, vitamin D needs to be metabolized into 25(OH)D in the liver, and subsequent into the active form 1,25-dihydroxyvitamin D [1,25(OH)2D] in the kidney before exerting its biological function (14). These processes request enzyme 25-hydroxylase, enzyme 1-α hydroxylase and vitamin D-binding protein, all of which are magnesium-dependent (14). Human studies also found that high magnesium intake was associated with reduced risk of vitamin D deficiency (19). In our study, we also performed multinomial logistic regression to explore the associations between low magnesium intake and serum 25(OH)D level (Supplementary Table S1). We found that low magnesium intake was positively associated with suboptimal serum 25(OH)D (OR, 1.61; 95% CI: 0.86–3.03; *P* = 0.131) and deficient serum 25(OH)D (OR, 1.58; 95% CI: 0.81–3.06; *P* = 0.171), but not statistically significant. The reason why these associations were non-significant might be that the sample size was relatively small. Considering the positive effect of serum vitamin D on muscle strength (20), low magnesium intake might link with grip strength *via* affecting the synthesis and transport of 1,25(OH)2D. Similar interactions between low magnesium intake and serum 25(OH)D level were observed on insulin resistance, hypertension, diabetes, and cardiovascular mortality (17–19, 40), but not on cognitive performance (41).

Our studies found that participants with both low magnesium intake and deficient serum 25(OH)D had highest decrease of handgrip strength. This finding suggested that increased intake of magnesium is especially needed for older people with deficient serum 25(OH)D in improving muscle strength.

Our studies had several strengths. Our study represents the first study to explore the associations of low magnesium intake with muscle strength in participants with different levels of serum 25(OH)D. In addition, participants of our study were from a nationally representative sample of US adults, which means our findings might be generalized to the whole US elderly people. Finally, we adjusted a series of known and potential confounders in the regression models.

There were also some limitations in our study. First, owning to the cross-sectional design, we were unable to explicate the causal relationship between low magnesium intake and handgrip strength. Second, magnesium intake was evaluated by using data from self-reported dietary recalls, which suggested that recall bias and reporting bias might exist in this study. Third, because of data availability, we were incapable of clarifying whether interactions also existed between serum magnesium and serum vitamin D level on muscle strength. Fourth, we could not explore the individual and interactive effects of magnesium intake and vitamin D on muscle mass and physical performance because related data were not collected among the elderly.

## Conclusion

In conclusion, the present study indicated that serum 25(OH)D level modified the associations between low magnesium intake and handgrip strength, and low magnesium intake was associated with reduced handgrip strength only in the elderly with deficient serum 25(OH)D. Increased magnesium intake was recommended for participants with deficient serum 25(OH)D in preventing muscle weakness. More prospective studies are needed to examine the causal effect between low magnesium intake and muscle strength.

## Data availability statement

Publicly available datasets were analyzed in this study. This data can be found here: https://www.cdc.gov/nchs/nhanes/.

## Ethics statement

The studies involving human participants were reviewed and approved by the National Center for Health Statistics Research Ethics Review Board. The patients/participants provided their written informed consent to participate in this study.

## Author contributions

SH, YG, and DG designed the study. SH analyzed the data and wrote the manuscript. YG and DG revised the manuscript and supervised the study. All authors read and approved the final version of the manuscript.

## Funding

This study was supported by grants from the National Natural Science Foundation of China (82100904), the Zhejiang Provincial Natural Science Foundation of China (LQ21C110001), the China Postdoctoral Science Foundation (2020M671696), and the Construction Fund of Key Medical Disciplines of Hangzhou (No. OO20200055).

## Conflict of interest

The authors declare that the research was conducted in the absence of any commercial or financial relationships that could be construed as a potential conflict of interest.

## Publisher's note

All claims expressed in this article are solely those of the authors and do not necessarily represent those of their affiliated organizations, or those of the publisher, the editors and the reviewers. Any product that may be evaluated in this article, or claim that may be made by its manufacturer, is not guaranteed or endorsed by the publisher.
