# Supplementary material for: Associations between dietary magnesium intake and handgrip strength were modified by serum vitamin D level among the US elderly
Source: Front Nutr. 2022 Oct 12;9:1002634. doi: 10.3389/fnut.2022.1002634 (PMC9596937; doi:10.3389/fnut.2022.1002634)
Supplement: Supplementary file 1 [file Table_1.DOCX]

Supplementary table S1. Associations between low magnesium intake and serum 25-hydroxyvitamin D level

|  |  | Serum 25(OH)D suboptimal | | |  | Serum 25(OH)D deficient | | |
| --- | --- | --- | --- | --- | --- | --- | --- | --- |
|  |  | OR (95% CI) | | *P* |  | OR (95% CI) | | *P* |
| Low magnesium intake |  |  |  |  |  |  |  |  |
| Crude model |  | 1.36 (0.86, 2.15) | | 0.184 |  | 1.83 (1.07, 3.13) | | 0.028 |
| Adjusted model |  | 1.61 (0.86, 3.03) | | 0.131 |  | 1.58 (0.81, 3.06) | | 0.171 |

Adjusted model: Adjusted for age, gender, BMI group, race, marital status, education, family monthly poverty level index category, smoke, alcohol drinking, physical activity, energy intake, percentage of total energy from protein, calcium intake, hypertension, diabetes, coronary heart disease, stroke, cancer, and season of examination.

25(OH)D, 25-hydroxyvitamin D; OR, odds ratio; CI, confidence interval.
